# Risk Assessment on Dietary Exposure to Aflatoxin B_1_ in Post-Harvest Peanuts in the Yangtze River Ecological Region

**DOI:** 10.3390/toxins7104157

**Published:** 2015-10-15

**Authors:** Xiaoxia Ding, Linxia Wu, Peiwu Li, Zhaowei Zhang, Haiyan Zhou, Yizhen Bai, Xiaomei Chen, Jun Jiang

**Affiliations:** 1 Oil Crops Research Institute, Chinese Academy of Agriculture Science, Wuhan 430062, China; E-Mails: dingdin2355@sina.com (X.D.); wulinxia89@126.com (L.W.); zhaowei_zhang@126.com (Z.Z.); zhouhaiyan@caas.cn (H.Z.); baiyizhen224@126.com (Y.B.); chenxiaomei_200870@126.com (X.C.); jiangjun@126.com (J.J.); 2 Laboratory of Quality & Safety Risk Assessment for Oilseed Products (Wuhan), Ministry of Agriculture, Wuhan 430062, China; 3 Key laboratory of Detection for Mycotoxins, Ministry of Agriculture, Wuhan 430062, China; 4 Key Laboratory of Biology and Genetic Improvement of Oil Crop, Ministry of Agriculture, Wuhan 430062, China; 5 Quality Inspection &Test Center for Oilseed Products, Ministry of Agriculture, Wuhan 430062, China

**Keywords:** aflatoxin B_1_, peanut, dietary exposure, Yangtze River ecological region, climate

## Abstract

Based on the 2983 peanut samples from 122 counties in six provinces of China’s Yangtze River ecological region collected between 2009–2014, along with the dietary consumption data in Chinese resident nutrition and health survey reports from 2002 and 2004, dietary aflatoxin exposure and percentiles in the corresponding statistics were calculated by non-parametric probability assessment, Monte Carlo simulation and bootstrap sampling methods. Average climatic conditions in the Yangtze River ecological region were calculated based on the data from 118 weather stations via the Thiessen polygon method. The survey results found that the aflatoxin contamination of peanuts was significantly high in 2013. The determination coefficient (*R*^2^) of multiple regression reflected by the aflatoxin B_1_ content with average precipitation and mean temperature in different periods showed that climatic conditions one month before harvest had the strongest impact on aflatoxin B_1_ contamination, and that Hunan and Jiangxi provinces were greatly influenced. The simulated mean aflatoxin B_1_ intake from peanuts at the mean peanut consumption level was 0.777–0.790 and 0.343–0.349 ng/(kg·d) for children aged 2–6 and standard adults respectively. Moreover, the evaluated cancer risks were 0.024 and 0.011/(100,000 persons·year) respectively, generally less than China’s current liver cancer incidence of 24.6 cases/(100,000 persons·year). In general, the dietary risk caused by peanut production and harvest was low. Further studies would focus on the impacts of peanut circulation and storage on aflatoxin B_1_ contamination risk assessment in order to protect peanut consumers’ safety and boost international trade.

## 1. Introduction

Aflatoxins (AFTs) are chemicals that are acutely and chronically toxic to human and animals. The four major naturally produced AFTs are aflatoxins B_1_, B_2_, G_1_, and G_2_ [1], among which aflatoxin B_1_ (AFB_1_) is generally the most prevalent and toxic [2]. AFTs in nature are produced mainly by *Aspergillus flavus* and *Aspergillus parasiticus*, which have a particular affinity to nuts and oilseeds. Peanuts are one of the most seriously affected crops because the seed-bearing pods of peanuts are below the soil surface and in direct contact with soil populations of *A. flavus* and *A. parasiticus*. *A. flavus* produces aflatoxins B_1_ and B_2_, and *A. parasiticus* produces aflatoxins G_1_ and G_2_ [3]. The seeds are often infected by both species before harvest. Agricultural practices including crop rotation, tillage, irrigation and fertilization, as well as planting date, genetic resistance, soil type and climatic conditions are all factors that impact AFT contamination of peanuts before harvest [4]. Nevertheless, climatic conditions significantly influence the AFT contamination level. In serious drought and/or high temperature conditions before harvest, fungi invasion and AFT accumulation become accelerated [5,6].

**Figure 1:**
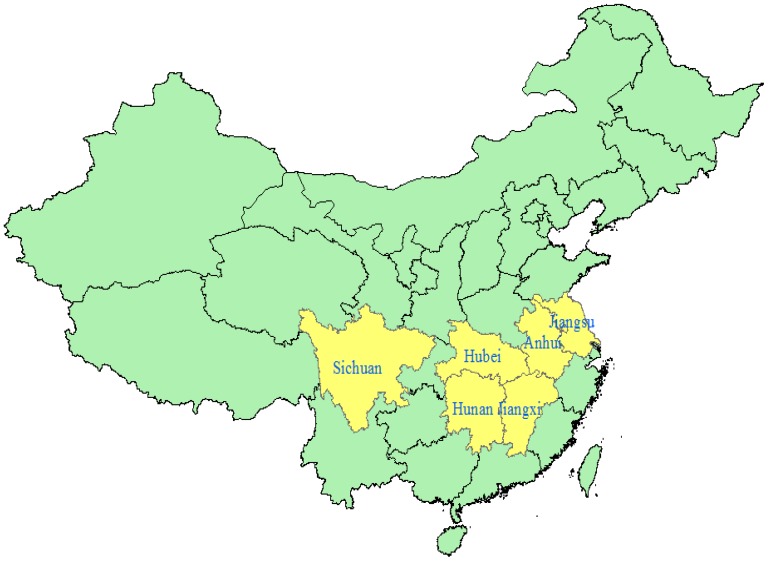
The six main peanut producing provinces in the Yangtze River ecological region in China.

According to different locations, terrain features, climatic conditions, variety distributions and cultivation systems, the peanut planting areas of in China were mainly classified into four sections: Northeast, North, Yangtze River and South [7]. The sowing areas and yields in the Yangtze River ecological region including Jiangsu, Anhui, Jiangxi, Hubei, Hunan, and Sichuan provinces (Figure 1) account for 1/5 of the whole country’s. However, AFT contamination is the highest in the Yangtze River area. Research has shown that AFTs are appropriately recognized as a cause of human liver cancer, and the cancer risk due to exposure to AFTs has been well established [8,9,10,11]. Research has also shown that the potency of AFTs in individuals positive for hepatitis B virus (HBsAg+) is substantially higher (about a factor of 30) than that in individuals negative for hepatitis B virus (HBsAg−) [12].

Risk assessment is an internationally recognized theoretical basis and technical support for quality and safety evaluation, standard establishment and risk management of agricultural products. However, no study on the risk assessment of AFB_1_ in peanuts in the Yangtze River ecological region has ever been conducted. Dietary exposure assessment and risk characterization are important components of risk assessment. Therefore, AFB_1_ assessment research is important for preparing scientific and efficient risk management measures, reducing dietary intake, and improving the peanut industry and international trade.

The aim of this work was to determine the prevalence of AFB_1_ in peanuts at harvest in the Yangtze River ecological region caused by the heaviest contamination in the hot and humid climate (especially in the plum rain season) in southern China; investigate the contamination reasons related to climatic conditions; and assess the safety risk posed to human diets based on the obtained results.

## 2. Results and Discussion

### 2.1. Validation of Chromatographic Methods

The detection limits of HPLC was 0.2 μg/kg, linear range was 0.8–60 μg/kg, correlation coefficient was 0.9998 and retention time of AFB_1_ was 14.5 min.

### 2.2. Contamination Distributions of AFB_1_ in 2009–2014

Based on the AFB_1_ data of 2983 samples collected from the six provinces of the Yangtze River ecological region in 2009–2014, AFB_1_ content distributions in peanuts were established; the statistical results are shown in Table 1. The results show that AFB_1_ contamination was not detected in 69% of the data. When the non-detected values were replaced by 0 and the limit of detection (LOD), the standard deviation and percentiles above P75 of the AFB_1_ content remained consistent. The mean value and percentiles below P70 of AFB_1_ were influenced by the method for estimating the non-detected values. The content difference was within the range of 0.20 μg/kg.

**Table 1:** Distribution statistics for the AFB_1_ content in peanuts in the Yangtze River ecological region (2009–2014).

Statistic	Methods	AFB_1_ Content (μg/kg)
**Mean**	LB ^a^	7.101
	UB ^a^	7.238
**Standard deviation**	LB	25.215
	UB	25.177
**P25**	LB	0.000
	UB	0.200
**P50**	LB	0.000
	UB	0.200
**P65**	LB	0.000
	UB	0.200
**P70**	LB	0.131
	UB	0.200
**P75**	LB	0.280
	UB	0.280
**P90**	LB	12.621
	UB	12.621
**P95**	LB	56.485
	UB	56.485
**P97.5**	LB	92.467
	UB	92.467

^a^ LB, values below the limit of detection (LOD) were replaced by zero; UB values below the LOD were replaced by the LOD.

### 2.3. Natural Occurrence of AFB_1_ in Post-Harvested Peanuts in the Yangtze River Ecological Region (2009–2014)

The results of the six-year survey for peanuts in the Yangtze River ecological region were summarized in Table 2. Among the 2983 samples directly collected from the fields, the percentage ofundetected-AFB_1_ (whose content is lower than the LOD) in six provinces was 69%. More than 82% of AFB_1_ or AFTs detected in the peanut samples was less than 1.0 μg/kg. According to the Chinese AFB_1_ regulation (<20 μg/kg), 8.55% of the samples exceeded the standard, demonstrating that AFB_1_ contamination in peanuts in the Yangtze River ecological region had occurred before harvest.

**Table 2:** AFB_1_ content in peanuts in the Yangtze River ecological region (2009–2014).

Year	Location	NO.	Positive Samples (%)	Mean (μg/kg)	Std. Deviation (μg/kg)	P90 (μg/kg)	Compliance ^a^ (%)
**2009**	Anhui	87	25 (28.74)	2.82	11.49	1.63	83 (95.40)
	Hubei	131	57 (43.51)	1.70	7.50	0.49	128 (97.71)
	Hunan	36	4 (11.11)	0.71	2.99	0.16	100
	Jiangxi	32	17 (53.13)	4.16	8.91	17.13	30 (93.75)
	Jiangsu	52	7 (13.72)	1.34	7.11	0.23	51 (98.08)
	Sichuan	-	-	-	-	-	-
	Total	339	110 (32.45)	2.06	8.48	1.41	329 (97.05)
**2010**	Anhui	82	9 (10.98)	4.48	19.49	3.41	77 (93.90)
	Hubei	93	63 (67.74)	3.18	11.87	4.29	90 (96.77)
	Hunan	70	30 (48.86)	3.00	13.49	2.08	68 (97.14)
	Jiangxi	92	37 (40.22)	6.59	22.70	12.43	85 (92.39)
	Jiangsu	60	0	0.00	0.00	0.00	100
	Sichuan	80	2 (2.5)	0.30	2.38	0.00	79 (98.75)
	Total	477	141 (29.56)	3.15	14.93	2.77	459 (96.23)
**2011**	Anhui	149	68 (45.64)	2.48	12.50	1.52	146 (97.99)
	Hubei	99	31 (31.31)	2.98	19.64	0.64	96 (96.97)
	Hunan	86	50 (58.14)	11.96	38.58	33.40	74 (86.05)
	Jiangxi	93	54 (58.06)	11.41	32.54	39.35	79 (84.95)
	Jiangsu	100	55 (0.55)	0.56	3.24	0.39	99 (99)
	Sichuan	97	21 (21.65)	8.37	29.33	4.82	88 (90.72)
	Total	624	279 (44.71)	5.80	24.71	4.76	612 (98.08)
**2012**	Anhui	150	32 (21.33)	2.94	11.86	2.02	143 (95.33)
	Hubei	47	10 (21.28)	2.33	6.65	7.96	45 (95.74)
	Hunan	140	33 (23.57)	15.49	45.59	48.56	118 (84.29)
	Jiangxi	130	44 (33.85)	6.49	15.30	21.76	115 (88.46)
	Jiangsu	100	14 (0.14)	0.44	2.89	0.20	99 (99)
	Sichuan	140	26 (18.57)	8.19	25.01	22.63	124 (88.57)
	Total	707	159 (22.49)	6.72	25.19	14.43	644 (91.09)
**2013**	Anhui	149	61 (40.94)	12.66	29.47	57.34	125 (83.89)
	Hubei	98	33 (33.67)	14.86	30.16	73.23	78 (81.63)
	Hunan	110	34 (30.91)	21.51	46.23	113.50	88 (80)
	Jiangxi	100	20 (20)	11.73	32.98	46.56	88 (88)
	Jiangsu	100	53 (53)	12.79	26.82	59.95	82 (82)
	Sichuan	140	21 (15)	7.41	26.24	8.59	128 (91.43)
	Total	697	222 (31.85)	13.20	32.55	60.46	589 (84.51)
**2014**	Anhui	79	22 (27.85)	17.14	43.11	81.08	67 (84.81)
	Hubei	17	0	0.00	0.00	0.00	100
	Hunan	7	0	0.00	0.00	0.00	100
	Jiangxi	24	2 (8.33)	2.36	7.98	0.00	22 (91.67)
	Jiangsu	12	0	0.00	0.00	0.00	100
	Sichuan	-	-	-	-	-	-
	Total	139	24 (17.27)	10.15	33.56	18.02	125 (89.93)

^a^ compliance-the rate of samples below the Chinese limit standard.

Compared with the incidence of AFB_1_ in peanuts found in previous reports conducted in other countries and regions (Table 3), the maximum AFB_1_ content in our study was higher. However, the high AFB_1_ values were mostly distributed in Jiangxi (2011) and Hunan provinces (2011, 2012). In addition, the Yangtze River ecological region is located at 24°–36°N and 97°–122°E, where the climatic conditions are in favor of AFT contamination. The regulatory limit for AFB_1_ contamination is 20 μg/kg in China. The mean content of AFB_1_ in São Paulo was low because of the compulsory good manufacturing practices (GMP) in the Brazilian peanut industry since 2003. The mean AFB_1_ content was also relatively low in Uganda, Korea and Taiwan. The regulatory limit is 10 μg/kg in Uganda, 10 μg/kg (AFB_1_) in Korea and 15 μg/kg (total AFT concentrations) in Taiwan.

**Table 3:** Recent reports on AFB_1_ in raw peanuts.

Country	Year Reported	Incidence (%)	Content (μg/kg)	Analysis Method
**Egyptian [13]**	-	82 (in peanuts and seeds)	24	-
**India [14]**	-	-	<833	HPLC ^a^
**Uganda [15]**	2003–2004	-	7.3–12.4	-
**Korea [16]**	2004–2005	53.33	4.07 (0.11–18.04)	HPLC
**São Paulo [17]**	1995–1996	31.43	<1557	TLC ^b^
**São Paulo [18]**	2006–2007	47.92	6.02	HPLC
**Congo [19]**	-	72	229.07 (1.5–937)	TLC
**Taiwan [20]**	1997–2011	7.8	1.56	HPLC
**Bulawayo [21]**	-	17	6.3–528	-

^a^ HPLC: high performance liquid chromatography; ^b^ TLC: thin layer chromatography.

### 2.4. Relationship between AFB_1_ Contamination Levels in Peanuts and Climatic Conditions before Harvest

**Figure 2:**
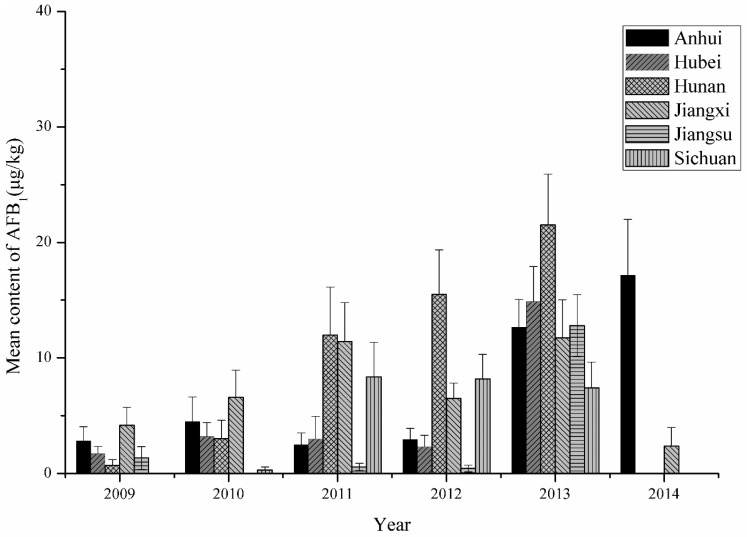
Mean contents of AFB_1_ in six provinces in the Yangtze River ecological region (2009–2014).

**Figure 3:**
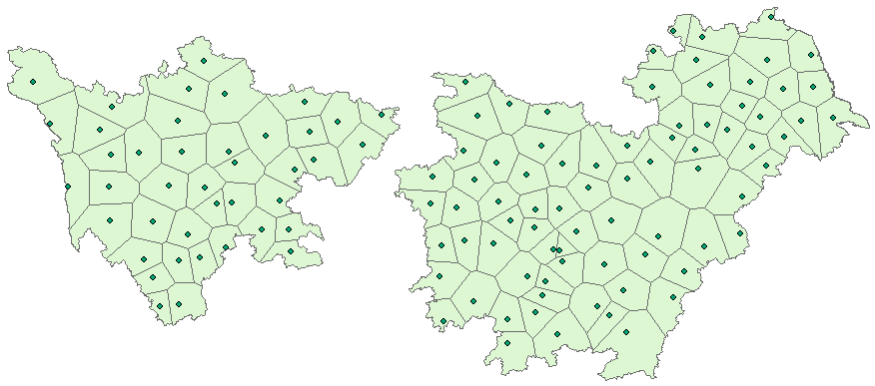
Data from 118 weather stations in the Yangtze River ecological region analyzed by Thiessen polygon interpolation.

**Figure 4:**
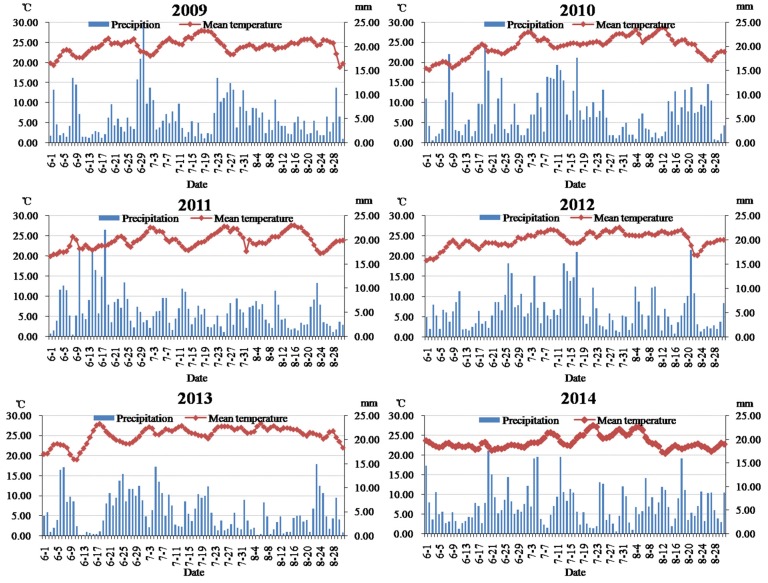
Precipitation and mean temperature of the Yangtze River ecological region during the peanuts’ growing season (2009–2014).

**Figure 5:**
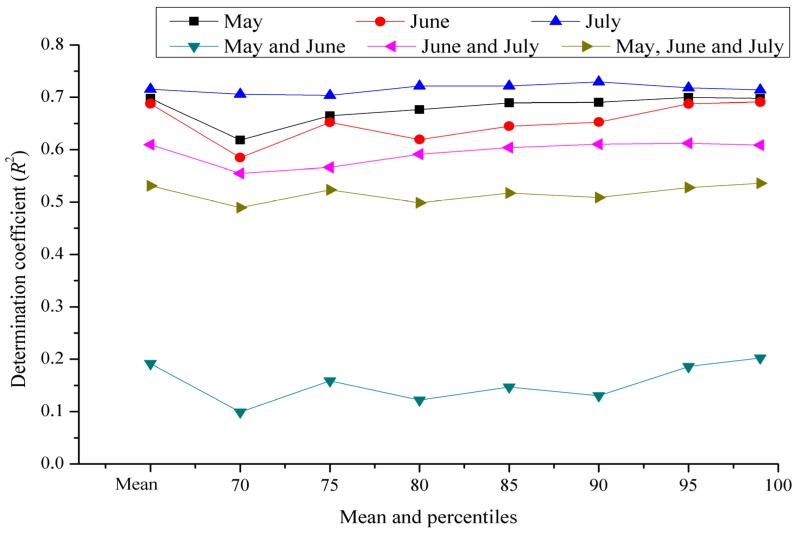
Multiple regression determination coefficient (*R*^2^) fitted by the AFB_1_ content with average precipitation and mean temperature in different periods of time (Hunan province).

Cole *et al.* (1985) found that apparently undamaged peanuts grown under the environmental stress with drought and heat (25.7–31.3 °C) became contaminated with *Aspergillus* *flavus* and AFT in the last 4–6 weeks of the growing season [22]. In this study, the multiple comparison results showed that AFB_1_ pollution in peanuts in 2013 was more serious in the Yangtze River ecological region (Figure 2). The analysis result by Thiessen polygon interpolation was illustrated in Figure 3, based on which the average climatic conditions of the Yangtze River ecological region could be obtained (Figure 4). In Figure 4, we noticed that precipitation was light and the daily mean temperature was high (around 25 °C) during the peanut growing season (June to August) in 2013, which favored AFB_1_ contamination in peanuts. Taking Hunan province where peanuts were harvested at late July or early August as an example, the determination coefficient (*R*^2^) of multiple regression fitted by the AFB_1_ content with average precipitation and mean temperature in different periods was presented in Figure 5. The results showed that the climatic conditions one month before harvest (July) had a strong impact on AFB_1_ contamination, and Hunan and Jiangxi were greatly influenced. It confirmed that the climate during peanut growing season had a significant impact on AFB_1_ contamination in post-harvest peanuts from another point of view. Because the AFB_1_ contamination level varied with year and climate, it is necessary to develop a consecutive and effective AFB_1_ monitoring program for pre-harvest peanuts and climatic conditions during its growing season to build an early warning model. There had been certain progress on model building in Australia and USA [23,24].

### 2.5. AFB_1_ Risk Assessment

#### 2.5.1. AFB_1_ Dietary Exposure Assessment

Based on the AFB_1_ contamination data of the peanut samples combined with the peanut consumption data and demographic data (Table 4), AFB_1_ intake via peanuts of different population groups was simulated and calculated. The distributions of the estimated intake percentiles (90% confidence interval) are shown in Table 5. Evenwithin the same region, the AFT-induced liver cancer risk varied significantly among different populations. The mean AFB_1_ intake through peanuts for 2- to 6-year-old children was higher than adults. Simulated AFB_1_ intake at the mean peanut consumption level ranges from 0.777 ng/(kg·d) (LB estimate) to 0.790 ng/(kg·d) (UB estimate), and at the high peanut consumption level it ranges from 11.660 ng/(kg·d) (LB estimate) to 11.853 ng/(kg·d) (UB estimate). The high percentile (P97.5) at the mean peanut consumption level ranges from 10.007 ng/(kg·d) (LB estimate) to 10.022 ng/(kg·d) (UB estimate), and at the high peanut consumption level it ranges from 150.104 ng/(kg·d) (LB estimate) to 150.330 ng/(kg·d) (UB estimate). The high AFT exposure for children and the effects of this exposure on children’s growth have been demonstrated in West Africa [25]. Because the AFB_1_ dietary exposure level in children is high and its influence on the high immunity level and different aspects of children’s health is significant [26], China should establish more strict regulations on the control of the processing conditions of peanuts, sorting techniques and so on to limit AFT at the consumer level.

**Table 4:** Peanut consumption groups and consumption amount.

Group	Weight/kg	Amount of Peanut Consumption/g	
		Mean-Level Consumption	High-Level Consumption
**2- to 6- year-old children**	15.18	1.66	24.9
**Standard adult ^a^**	62.57	3.02	35.7

^a^ Adult male who engages in light physical labor.

#### 2.5.2. AFB_1_ Risk Characterization

Based on AFB_1_ intake in the above two population groups through peanuts and risk assessment method established by Joint FAO/WHO Expert Committee on Food Additives (JECFA) [27], liver cancer risk related to AFB_1_ dietary exposure through peanuts and an average potency figure obtained from the individual potencies of hepatitis B surface antigen-positive and -negative groups were evaluated by JECFA. In China, the hepatitis B prevalence rate is assumed to be 7.18%. The cancer risk characterization results are shown in Table 6. In light of the national average HBsAg+ prevalence rate, even at the high risk level of P97.5, the liver cancer risk (90% confidence interval) resulting from peanut AFB_1_ exposure in adults with high peanut consumption ranged from 1.619 (1.524–1.692) cases/(100,000 persons·year) (LB estimate) to 1.621 (1.524–1.693) cases/(100,000 persons·year) (UB estimate), less than China’s current liver cancer incidence of 24.6 cases/(100,000 persons·year) [28]. Research showed that liver cancer is a disease prevalent in some developing parts of the world, such as China, South East Asia and sub-Saharan Africa [27]. Harris *et al.* (1996) reviewed the evidence that “a dose-dependent relationship between dietary AFB_1_ intake and p53 mutations (codon 249 ser) is observed in hepatocellular carcinoma from Asia, Africa and North America” [29]. Therefore, the heavily-contaminated regions of AFB_1_ are in good agreement with liver cancer prevalence regions. Due to high AFB_1_ intake by 2- to 6-year-old children with high peanut consumption, their liver cancer risk was relatively high and noteworthy, and essential surveillance measures should be taken to protect their health.

Generally, based on the raw peanut samples from the post-harvest fields in the Yangtze River ecological region, dietary exposure assessment results indicate that the occurrence of AFB_1_ in raw peanuts at harvest does not appear to be a serious problem and that the risk concerning public health is low in China.

Different countries have varied AFB_1_ exposure levels. Data assembled in the researches indicated that the exposure to AFTs through frequently contaminated foods was 3.5–14.8 ng/(kg·d) in Kenya, 11.4–158.6 ng/(kg·d) in Swaziland, 38.6–183.7 ng/(kg·d) in Mozambique, 16.5 ng/(kg·d) in Transkei (now South Africa), and 4–115 ng/(kg·d) in Gambia. The AFTs exposure in Ghana, as measured from peanut consumption alone, is estimated to be 9.9–99.2 ng/(kg·d). The estimated mean AFB_1_ exposure for urban Lebanese adults was 0.63–0.66ng/(kg·d) and P95 was 1.40–1.46ng/(kg·d). Based on the mean dietary exposure level of AFB_1_, the cancer risk was estimated to be0.0527–0.0545 cases/(100,000 persons·year) [30]. As for Asia, the estimated AFTs intake was 11.7–2027ng/(kg·d) in southern Guangxi province of China and 6.5–53 ng/(kg·d) in Thailand.Sugita-Konishi *et al.* (2010) assessed AFTs dietary exposure by food intake in different age groups, and the results suggested that the dietary intake of AFB_1_ ranged from 0.003 to 0.004 ng/(kg·d) (from lower to upper limits), and the potential cancer risk was 0.00004–0.00005 cases/(100,000 persons·year) persons in the high level (95.0th percentile) of the consumer population. The mean dietary intake of AFB_1_ through peanuts was 0.49ng/(kg·d) [31]. However, the European Union estimated that the dietary exposure to AFTs ranged from 0.93 to 2.45 ng/(kg·d) for lower bound to upper bound [27,32]. In the United States, the exposure was estimated at 2.7 ng/(kg·d) [8]. The AFB_1_ intake estimates in our study were relatively high because peanuts were one of the products most likely affected by AFTs. Therefore, it is very important to regulate and monitor AFT contamination to protect peanut consumers, especially children and vegans. The results agreed with Wilda *et al.*’s findings (2000) that the most heavily AFT-afflicted parts of the world were sub-Saharan Africa, Southeast Asia, and China [33]. On the one hand, the reason may lie in the particularly high risk areas in tropical and subtropical regions and exposure to climatic conditions favorable for *A. flavus* and *A. parasiticus* proliferation. On the other hand, limited AFT control strategies were implemented in these countries. Prediction before harvest, good agricultural practice (GAP), good manufacturing practice (GMP), hazard analysis and critical control point (HACCP) are all effective measures for AFT control in peanuts.

Ding *et al.* (2012) indicated that the risk from peanut oil was about ten times than from raw peanuts in China. Consequently, AFB_1_ control for post-harvest products including storage conditions, processing methods and so on is critical to aggravate AFB_1_ contamination in the Yangtze River ecological region, besides growing and harvest. Further studies need to be focused on the process of peanut circulation and storage for AFB_1_ contamination risk assessment.

**Table 5:** Simulated AFB_1_ intake through peanuts in different population groups in the Yangtze River ecological region.

Population	Consumption Level	Methods	Mean (90% Confidence Interval)/ng/(kg·d)	Percentiles of AFB_1_ Intake (90% Confidence Interval)/ng/(kg·d)				
				P50	P75	P90	P95	P97.5
**2- to 6-year-old child**	Mean	LB ^a^	0.777 (0.729–0.825)	0	0.031 (0.028–0.034)	1.377 (1.230–1.501)	6.131 (5.796–6.383)	10.007 (9.423–10.462)
		UB ^a^	0.790 (0.745–0.837)	0.022	0.031 (0.028–0.035)	1.384 (1.231–1.503)	6.144 (5.796–6.383)	10.022 (9.423–10.465)
	High	LB	11.660 (10.934–12.370)	0	0.462 (0.427–0.509)	20.655 (18.454–22.509)	91.972 (86.937–95.751)	150.104 (141.338–156.929)
		UB	11.853 (11.174–12.556)	0.328	0.463 (0.427–0.509)	20.753 (18.470–22.538)	92.162 (86.937–95.751)	150.330 (141.338–156.972)
**Standard adult**	Mean	LB	0.343 (0.322–0.364)	0	0.014 (0.013–0.015)	0.608 (0.544–0.662)	2.706 (2.558–2.818)	4.417 (4.159–4.618)
		UB	0.349 (0.329–0.370)	0.010	0.014 (0.013–0.015)	0.611 (0.543–0.663)	2.712 (2.558–2.818)	4.423 (4.159–4.619)
	High	LB	4.056 (3.803–4.303)	0	0.161 (0.148–0.177)	7.185 (6.419–7.829)	31.991 (30.240–33.306)	52.212 (49.162–54.586)
		UB	4.123 (3.887–4.367)	0.114	0.161 (0.148–0.177)	7.219 (6.425–7.840)	32.057 (30.240–33.306)	52.290 (49.162–54.601)

^a^ LB, values below the limit of detection (LOD) were replaced by zero; UB values below the LOD were replaced by the LOD.

**Table 6:** Estimated liver cancer risk caused by AFB_1_ intake through peanuts in different population groups in the Yangtze River ecological region.

Population	Consumption Level	Methods	Mean (90% Confidence Interval)/cases/(10^5^·persons·year)	Percentiles of AFB_1_-Induced Liver Cancer Risk (90% Confidence Interval)/cases/(100,000·persons·year)				
				P50	P75	P90	P95	P97.5
**2- to 6-year-old child**	Mean	LB ^a^	0.024 (0.023–0.026)	0	0.001	0.043 (0.038–0.047)	0.190 (0.180–0.198)	0.310 (0.292–0.324)
		UB ^a^	0.024 (0.023–0.026)	0.001	0.001	0.043 (0.038–0.047)	0.190 (0.180–0.198)	0.311 (0.292–0.324)
	High	LB	0.361 (0.339–0.383)	0	0.014 (0.013–0.016)	0.640 (0.572–0.698)	2.851 (2.695–2.968)	4.653 (4.381–4.865)
		UB	0.367 (0.346–0.389)	0.010	0.014 (0.013–0.016)	0.643 (0.573–0.699)	2.857 (2.695–2.968)	4.660 (4.381–4.866)
**Standard adult**	Mean	LB	0.011 (0.010–0.011)	0	0	0.019 (0.017–0.021)	0.084 (0.079–0.087)	0.137 (0.129–0.143)
		UB	0.011 (0.010–0.012)	0	0	0.019 (0.017–0.021)	0.084 (0.079–0.087)	0.137 (0.129–0.143)
	High	LB	0.126 (0.118–0.133)	0	0.005 (0.004–0.006)	0.223 (0.199–0.243)	0.992 (0.937–1.032)	1.619 (1.524–1.692)
		UB	0.128 (0.120–0.135)	0.004	0.005	0.224 (0.199–0.243)	0.994 (0.937–1.032)	1.621 (1.524–1.693)

^a^ LB, values below the limit of detection (LOD) were replaced by zero; UB values below the LOD were replaced by the LOD.

## 3. Experimental Section

### 3.1. Samples

A total of 2983 raw peanut samples (in shell) were collected at harvest time from farmers’ fields in 122 counties of six provinces (Jiangsu, Anhui, Jiangxi, Hubei, Hunan, and Sichuan) in the Yangtze River ecological region between 2009–2014 by simple random sampling. Samples of at least 3.0 kg, included 10–20 samples from different villages and were collected yearly from each county. All samples were delivered to the laboratory in sealed bags and stored under ventilated and dry conditions. After impurities, dusts and shells were removed, the seeds were cut into 0.5 cm thick slices and ground, and then thoroughly mixed in a sample grinder until they could pass through a 0.9 mm sieve. The ground samples were put in glass containers and stored at 4 °C until analyzed by high performance liquid chromatography(HPLC) (NYSE: A, Palo Alto, CA, USA). All of this work was completed within 4 weeks.

### 3.2. Climate Data

The precipitation and mean temperature data between 2009–2014 were collected from the data set of land-based daily climate data in China Meteorological Data Sharing Service System, which was provided by 118 weather stations in the Yangtze River ecological region, including 14 weather stations in Anhui, 17 in Hubei, 23 in Hunan, 11 in Jiangsu, 12 in Jiangxi and 41 in Sichuan. The climatic data span a period from May to August, which is the main growing season of peanuts in the Yangtze River ecological region.

### 3.3. Determination of AFB_1_ via HPLC Analysis

Samples were purified by immunoaffinity columns (Youni Biotechnology Co., Ltd., Shanghai, China) and analyzed for AFB_1_ by HPLC with fluorescence detection according to the Chinese method standards [34] and AOAC method 994.08 [35] with certain modifications. Finely ground samples (5.0 g) were extracted with 15 mL of 4% NaCl-methanol-water. After ultrasonic extraction (50 °C, 10min) and filtration with double-layer slow quantitative filter paper, the filtrate (3 mL) was collected in a 50 mL centrifugal tube and then mixed with 8 mL of double distilled water (dd H_2_O). The mixture was kept overnight. 3 mL of supernatant was collected and diluted with 8 mL of pure water. The obtained 8 mL extracts were passed through AFT immunoaffinity columns with a flow rate of one droplet every second and then eluted with 1 mL methanol into test tubes. The eluates were filtered with organic membrane (0.22 μm) into glass tubes and prepared for HPLC quantitative analysis.

HPLC analysis was performed on an Agilent 1100 HPLC system (NYSE: A, Palo Alto, CA, USA) equipped with a fluorescence detector at wavelengths of 360 nm and 440 nm for excitation and emission, respectively. Chromatographic separation was performed on a Capcell Pak C-18 column (4.6 mm × 150 mm × 5 µm), with a water-methanol (55:45) mobile phase pumped at a constant flow rate of 0.7 mL/min and the injection volume being 10 µL.

### 3.4. Statistical Analysis

**Figure 6:**
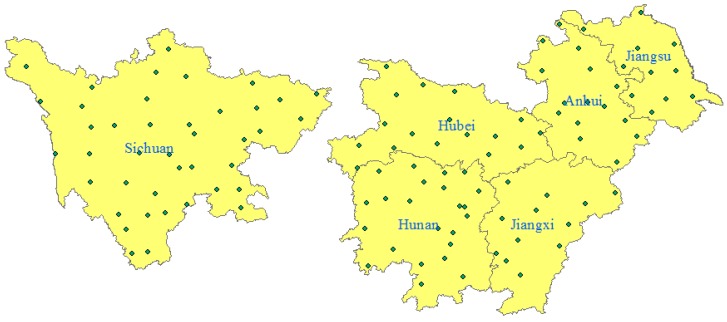
Data from 118 weather stations in the Yangtze River ecological region.

Average precipitation and mean temperature data from the 118 weather stations (Figure 6) of the Yangtze River ecological region in 2009–2014 were calculated by the Thiessen polygon method utilizing the ArcGIS 10.2 software (ArcGIS 10.2 for desktop; Esri, CA, USA).

The relationship between the AFB_1_ contents and climatic conditions is fitted by multiple linear regression with the SPSS software (IBM SPSS Statistics Version 20, IBM Corporation, Armonk, NY, USA), which is modeled by the following formula:

(1) $$Y=b_{1}+b_{2}\times Pr+b_{3}\times T^{2}+b_{4}\times T$$

*Pr* represents precipitation (mm), and *T* stands for mean temperature (°C).

### 3.5. Population Consumption Data

The dietary consumption data were obtained from the Chinese resident nutrition and health survey reports in 2002 and 2004 [36,37]. For children aged 2 to 6 years whose weight is 15.18 kg, the mean and high-level peanut consumption amounts are 1.66 g and 24.9 g, respectively. For male adults who engage in light physical labor weighed 62.57 kg, the mean and high-level peanut consumption amounts are 3.02 g and 35.7 g, respectively.

### 3.6. Risk Assessment

Distribution statistics for the estimated intake of AFB_1_ based on the Monte Carlo simulation and bootstrap sampling methods were performed with the @Risk program (@Risk for Excel 5.5.0 Industrial edition; Palisade Corporation, Sydney, Australia). According to the instructions of the Global Environment Monitoring System-Food Contamination Monitoring and Assessment Program, the values of the non-detected analytical results were calculated on the supposition that when the proportion of non-quantified or non-detected results was between 60% and 80%, the values under the LOD were replaced with zero or the LOD to produce lower and upper boundaries [38]. 10,000 iterations and 1000 simulations were conducted to estimate dietary exposure. The daily intake of AFB_1_ was calculated using the following formula:

(2) $$\text{Exposure}(\text{ng}/\text{kg})=\frac{\text{contamination level}(\text{μg}/\text{kg})\times \text{amount consumed}(\text{g})}{\text{body weight}(\text{kg})}$$

To estimate the risk posed by dietary exposure to AFB_1_, an excess risk model was simulated as follows:

(3) Population risk=Exposure×Average potency

(4) Average potency=0.3×P+0.01×(1−P)

*P* represents the hepatitis-B-virus surface antigen (HBsAg+) prevalence rate for different age groups. The final results included 10,000,000 simulations. The uncertainty of sampling was shown by the 90% confidence interval (90% CI, P5~P95) of the mean value, the percentiles and so on.

## 4. Conclusions

Based on a total of 2983 raw peanut samples (in shell) collected at harvest time by the simple random sampling method from farmers’ fields in six provinces (Jiangsu, Anhui, Jiangxi, Hubei, Hunan, and Sichuan) in the Yangtze River ecological region from 2009 to 2014, this study reported the natural occurrence of AFB_1_, the relationship between AFB_1_ contamination levels in peanuts and climatic conditions before harvest, as well as AFB_1_ dietary exposure and corresponding liver cancer risks in post-harvested peanuts. The AFB_1_ contamination in peanuts was significantly high in 2013, correlated with less precipitation and relatively high temperature in the month before harvest in Hunan and Jiangxi provinces, where the peanuts were significantly influenced. The mean AFB_1_ intake in 2- to 6-year-old children through peanuts was higher than adults. The estimated mean cancer risks in different population groups at the mean peanut consumption level were 0.024 and 0.011 cases/(100,000 persons·year) for 2- to 6- year-old children and adults, which were less than China’s current liver cancer incidence of 24.6 cases/(100,000 persons·year). In general, the post-harvest peanut dietary risk caused by the peanut production and harvesting process was low. Further studies would focus on the process of peanut circulation and storage for AFT contamination risk assessment. It is predicted to be significant to reduce the dietary risk of peanut AFTs by promoting an appropriate and balanced diet in addition to avoiding long-term and substantial peanut consumption.
